# A Case Report of Epidermodysplasia Verruciformis: Clinical Presentation and Histopathological Features

**DOI:** 10.7759/cureus.65249

**Published:** 2024-07-24

**Authors:** Abdullah A Alenazi, Bushra Alraddadi, Assaf G Alharbi

**Affiliations:** 1 Dermatology, Security Forces Hospital Program, Riyadh, SAU; 2 Dermatology, Ohud Hospital, Ministry of Health, Madina, SAU

**Keywords:** skin warts, ­skin cancer, epidermodysplasia verruciformis, hyperkeratosis, human papillomavirus (hpv)

## Abstract

Epidermodysplasia verruciformis (EV) is a rare, lifelong, autosomal recessive genodermatosis characterized by susceptibility to certain human papillomavirus (HPV) types and increased risk of skin cancer. This report describes a 22-year-old male presenting with multiple flat erythematous papules on the trunk and extremities. Histopathological examination of a skin biopsy revealed features consistent with EV, including hypergranulosis, hyperkeratosis, and acanthosis, with notable keratohyalin granules and perinuclear vacuolization of keratinocytes. No mitotic activity or cellular atypia was observed. This case underscores the importance of early diagnosis and management of EV, which includes genetic counseling, photoprotection, and regular monitoring for premalignant lesions. Treatment options, ranging from pharmacologic interventions to surgical excision, aim to mitigate the risk of malignant transformation. This report highlights the clinical and histopathological presentation of EV, contributing to the understanding and management of this rare genodermatosis.

## Introduction

The immune system is involved in the rare, lifelong, autosomal recessive genodermatosis known as epidermodysplasia verruciformis (EV). Initially appearing on the face, dorsum of the hands, and legs in early infancy, it is clinically defined by a spectrum of wart-like verrucous lesions and pityriasis versicolor-like patches [1]. However, in patients with EV, certain human papillomavirus (HPV) types may result in the formation of flat wart-like lesions that have the potential to advance to malignancy [2]. Over 30 forms of HPV, including HPV5, HPV8, HPV12, HPV14, HPV15, HPV17, HPV19-HPV25, HPV36, HPV38, HPV47, and HPV50, have been linked to EVs [1]. While HPV5 and HPV8 are recognized to have the potential to be cancerous, these HPV types are exclusively harmful in EV patients, although they may also be found in the general population [3].

## Case presentation

**Figure 1:**
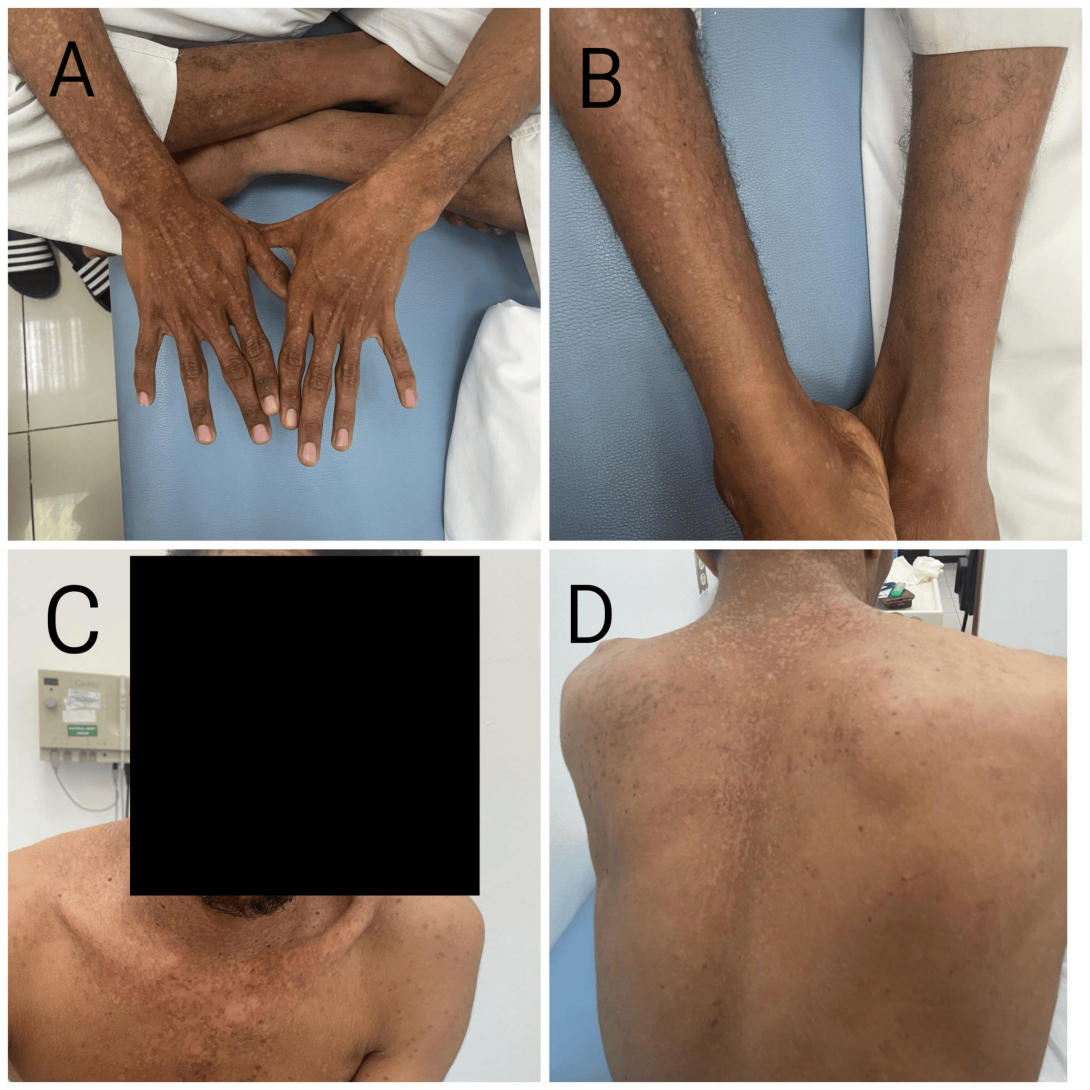
Epidermodysplasia verruciformis A, B, C, and D show multiple flat erythematous papules over the trunk and extremities.

A 22-year-old male patient presented with multiple flat, erythematous papules on the trunk and extremities (Figure 1). These lesions first appeared five years ago when the patient noticed small, flat, wart-like lesions on sun-exposed areas such as the face, neck, hands, and forearms. Over time, the number and size of the lesions increased, spreading to other areas such as the trunk and lower legs. Upon examination, multiple flat, erythematous papules were predominantly present on the trunk and extremities. Clusters of reddish-brown macules and patches resembling pityriasis versicolor were noted on the trunk and back. The lesions were light brown to pinkish in color and slightly scaly. The patient had no significant medical or surgical history, and there was no family history of any dermatological disease.

**Figure 2:**
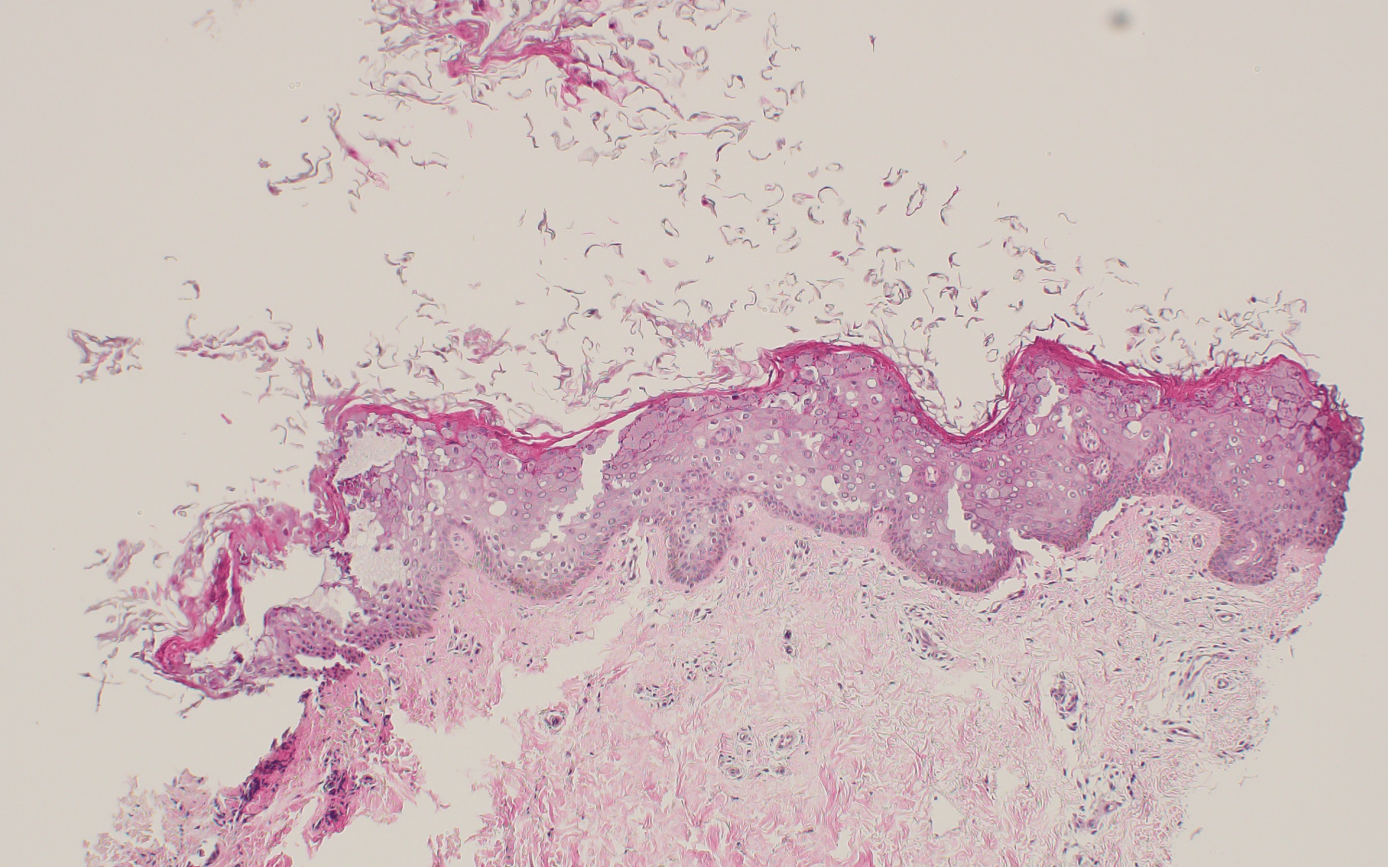
Mild to moderate acanthosis (low power) (H&E stain) H&E: hematoxylin and eosin

**Figure 3:**
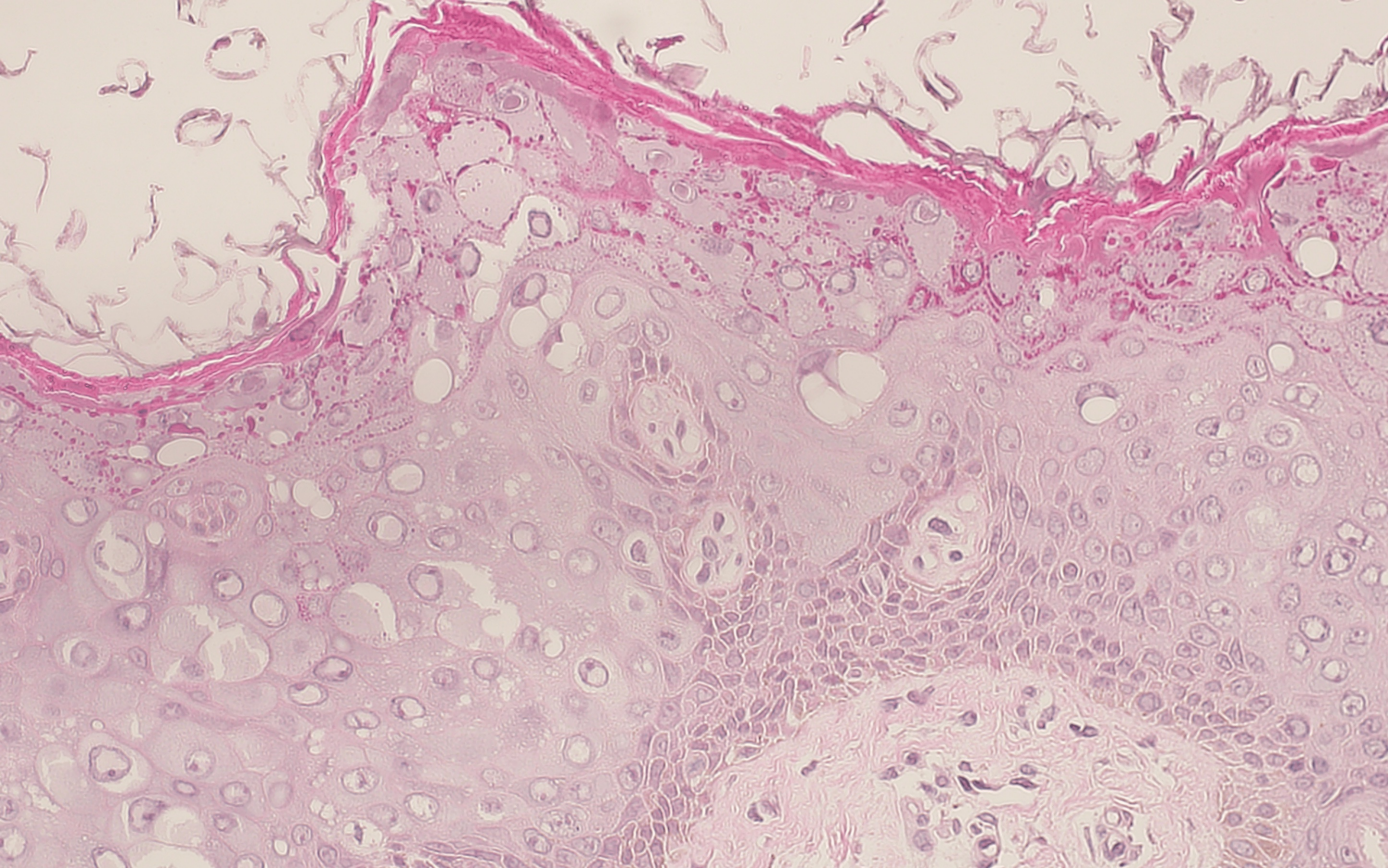
Large keratinocytes with abundant blue-gray cytoplasm and keratohyalin granules (H&E stain) H&E: hematoxylin and eosin

In light of the clinical presentation, a skin biopsy was taken from a representative lesion on the patient's back using a 3 mm punch biopsy. The specimen was sent for histological analysis. Microscopic examination of the biopsy material (Figure 2, Figure 3) revealed uneven hypergranulosis, hyperkeratosis, and noticeable acanthosis, all indicative of epidermal dysplasia. The upper layers of the epidermis showed prominent keratohyalin granules, clusters of larger keratinocytes with perinuclear vacuolization, and a distinctive bluish-gray cytoplasmic appearance. Importantly, there was no evidence of mitotic activity or notable cellular atypia upon thorough inspection. Based on the histological and clinical findings, a definitive diagnosis of epidermodysplasia verruciformis (EV) was made.

## Discussion

Epidermodysplasia verruciformis is a lifelong condition, which is asymptomatic in people of any gender, race, or location. It is a rare autosomal recessive genodermatosis that is distinguished by a higher risk of skin cancer [4]. The rarity of this disease can be estimated by the fact that until 2007, only 500 cases have been reported globally [5].

As was previously indicated, the disease may run in families or be sporadic. Nonetheless, two examples of the autosomal dominant pattern of inheritance have been documented [6]. In individuals with impaired immune systems, such as those undergoing kidney transplantation, Hodgkin's illness, systemic lupus erythematosus, and human immunodeficiency virus (HIV) infection, epidermodysplasia verruciformis may develop and is typically seen [4].

Mutations in the *EVER1* or *EVER2* genes, which are found on chromosome 17q25, cause abnormally high sensitivity in EV patients to a particular subset of HPV genotypes known as EV HPV (HPV5 and HPV8) [7]. This is due to a malfunction in cell-mediated immunity. Zinc transporter proteins play a part in inhibiting EV production, and a complex of EVER proteins regulates intracellular zinc homeostasis [8]. The hallmark of EV is a distinct impairment in cell-mediated immunity, as shown by the suppression of endogenous cytotoxicity and the growth of T lymphocytes directed against HPV-positive squamous cells inside EV skin lesions [9]. X-rays and ultraviolet (UV) B radiation are significant carcinogenic cofactors as well. In addition to immunologic deficiencies in EV patients, frequent sun exposure is likely to cause mutations in the tumor suppressor gene protein (p53), which causes skin cancer to develop in adult patients [10]. At about 40-50 years of age, 35%-50% of patients have transformation into malignancy; metastasis is rare and most often involves invasive squamous cell carcinoma after Bowen's-type carcinoma in situ [11]. The infecting virus' oncogenic potential is primarily responsible for its transformation into skin cancer. A young female was diagnosed with squamous cell carcinoma after presenting with a left infraorbital lesion [11].

Primary skin lesions often take the shape of flat-topped, lichenoid, warty papules. The lesions initially resemble tinea versicolor and are limited to the face and neck as hypochromic scaly patches [8]. They proliferate and eventually give rise to smooth-surfaced, pink-to-brownish papules that resemble flat warts. These papules measure a few millimeters and have a smooth surface. Later, they spread to the dorsum of the hands, wrists, knees, legs, and feet [8]. Certain lesions may disappear as the illness progresses, and other lesions may appear on different body areas. Results are limited to skin and seldom show up on mucous membranes. The patient's histological results in this case include epidermal dysplasia with acanthosis, hyperkeratosis, and irregular hypergranulosis. Furthermore, the diagnosis is supported by the observation of bluish-gray cytoplasmic appearance, abundant keratohyalin granules, and larger keratinocytes with perinuclear vacuolization.

Cimetidine, immunotherapy, imiquimod, interferon, oral and topical retinoids, and immunotherapy are examples of pharmacologic therapies [11]. The recommended medication at this time is acitretin, 0.5-1 mg daily [6]. When oral retinoids (0.5 mg/kg) were administered to two sisters with an autosomal recessive pattern, the size of the cutaneous lesions diminished quickly; however, a year later, the lesions relapsed [3]. Benign and premalignant lesions are also treated surgically, with electrosurgical excision and cryotherapy included [8]. Also treated surgically were malignant tumors. Surgery was used to treat squamous cell carcinoma on the face in an EV patient, and the results were better. Another case was the use of zinc treatment to treat EV, showing a 20%-40% response rate and no recurrence after six months [12].

As was said, there are a variety of treatment options available for EV; still, patient education, prompt diagnosis, and removal of premalignant and malignant lesions are the most crucial steps.

## Conclusions

To conclude, even in the absence of a family history, our case emphasizes the significance of diagnosing epidermodysplasia verruciformis (EV) in individuals presenting with distinctive skin lesions. This uncommon genodermatosis increases the risk of skin cancers since it is linked to HPV infection and weakened immunity. Through genetic counseling, photoprotection, and routine follow-up, management options concentrate on the prevention and early diagnosis of premalignant lesions. Retinoids, interferon, immunotherapy, and surgical procedures for both benign and malignant tumors are available forms of treatment.
